# Serotonin Heteroreceptor Complexes and Their Integration of Signals in Neurons and Astroglia—Relevance for Mental Diseases

**DOI:** 10.3390/cells10081902

**Published:** 2021-07-27

**Authors:** Dasiel O. Borroto-Escuela, Patrizia Ambrogini, Manuel Narvaez, Valentina Di Liberto, Sarah Beggiato, Luca Ferraro, Ramon Fores-Pons, Jose E. Alvarez-Contino, Alexander Lopez-Salas, Giuseppa Mudò, Zaida Díaz-Cabiale, Kjell Fuxe

**Affiliations:** 1 Department of Neuroscience, Karolinska Institutet, 171 77 Stockholm, Sweden; rforespons@gmail.com (R.F.-P.); erickjose@infomed.sld.cu (J.E.A.-C.); alexanderlopezsalas@gmail.com (A.L.-S.); 2 Department of Biomolecular Science Section of Morphology, Physiology and Environmental Biology, Campus Scientifico Enrico Mattei, via Ca’ le Suore 2, 61029 Urbino, Italy; patrizia.ambrogini@uniurb.it; 3 Facultad de Medicina, Instituto de Investigacion de Málaga, Universidad de Malaga, Campus de Teatinos s/n, 29071 Málaga, Spain; mnarvaez@uma.es (M.N.); zaida@uma.es (Z.D.-C.); 4 Department of Biomedicine, Neuroscience and Advanced Diagnostic (BIND), University of Palermo, 90134 Palermo, Italy; valentina.diliberto@unipa.it (V.D.L.); giuseppa.mudo@unipa.it (G.M.); 5 Department of Medical, Oral and Biotechnological Sciences, University of Chieti-Pescara, 66100 Chieti, Italy; sara.beggiato@unich.it; 6 Department of Life Sciences and Biotechnology and LTTA Center, University of Ferrara, 44121 Ferrara, Italy; frl@unife.it; 7 Department of Education, School of Medicine, Villa Clara University of Medical Sciences, Polyclinic Juan Bruno Zayas, 52900 Cifuentes, Cuba

**Keywords:** serotonin receptors, heteroreceptor complexes, depression, astroglia, receptor tyrosine kinase, rapid antidepressant drugs, G protein-coupled receptors

## Abstract

The heteroreceptor complexes present a novel biological principle for signal integration. These complexes and their allosteric receptor–receptor interactions are bidirectional and novel targets for treatment of CNS diseases including mental diseases. The existence of D2R-5-HT2AR heterocomplexes can help explain the anti-schizophrenic effects of atypical antipsychotic drugs not only based on blockade of 5-HT2AR and of D2R in higher doses but also based on blocking the allosteric enhancement of D2R protomer signaling by 5-HT2AR protomer activation. This research opens a new understanding of the integration of DA and 5-HT signals released from DA and 5-HT nerve terminal networks. The biological principle of forming 5-HT and other heteroreceptor complexes in the brain also help understand the mechanism of action for especially the 5-HT hallucinogens, including putative positive effects of e.g., psilocybin and the indicated prosocial and anti-stress actions of MDMA (ecstasy). The GalR1-GalR2 heterodimer and the putative GalR1-GalR2-5-HT1 heteroreceptor complexes are targets for Galanin N-terminal fragment Gal (1–15), a major modulator of emotional networks in models of mental disease. GPCR-receptor tyrosine kinase (RTK) heteroreceptor complexes can operate through transactivation of FGFR1 via allosteric mechanisms and indirect interactions over GPCR intracellular pathways involving protein kinase Src which produces tyrosine phosphorylation of the RTK. The exciting discovery was made that several antidepressant drugs such as TCAs and SSRIs as well as the fast-acting antidepressant drug ketamine can directly bind to the TrkB receptor and provide a novel mechanism for their antidepressant actions. Understanding the role of astrocytes and their allosteric receptor–receptor interactions in modulating forebrain glutamate synapses with impact on dorsal raphe-forebrain serotonin neurons is also of high relevance for research on major depressive disorder.

## 1. Introduction

The concept of allosteric receptor–receptor interactions in which G protein-coupled receptor (GPCR) homo-and heteroreceptor complexes, physically interact with each other and adaptor proteins, provides a new dimension to molecular integration in the central nervous system (CNS). Interactions through allosteric mechanisms dynamically alter recognition, pharmacology, signaling, and trafficking of the receptor protomers that include e.g., also ionotropic receptors and receptor tyrosine kinases (RTKs). Allosteric mechanisms come together with phosphorylation and dephosphorylation mechanisms to optimize the molecular integration of dynamic receptor complexes located in the synaptic and extra-synaptic membranes and necessary for CNS function. The heteroreceptor complexes present a novel biological principle for signal integration. Their allosteric receptor–receptor interactions are bidirectional and the complexes represent novel targets for treatment of CNS diseases [1]. The serotonin (5-HT) heteroreceptor complexes appear to have a significant role in major depression [2,3].

Selective serotonin reuptake inhibitor (SSRIs) antidepressants have had only moderate success with only one-third of the patients responding well to the treatment [4,5]. It may be related to the problem that increases in extracellular serotonin levels induced by SSRIs, activates not only 5-HTR subtypes that reduce depression (such as postsynaptic 5-HT1AR and 5-HT4R) but also others that enhance depression (such as 5-HT1A auto-receptors and 5HT2A/C receptors) [3,5,6]. However, there exist new opportunities to enhance the actions of SSRIs based on the existence of serotonin heteroreceptor complexes in which different subtypes of 5-HT receptors, dopamine (DA) receptors, RTKs, and neuropeptide receptors such as galanin receptor 1 and galanin receptor 2 (GalR1-GalR2) can participate [2,6,7,8,9,10].

In this article we, in part, focus on the role of the 5-HT2A-D2R [11,12] and putative GalR1-GalR2-5-HT1AR heterocomplexes in mental disease, with a particular focus on major depression [9]. The impact of GPCR-RTK heterocomplexes will also be covered. Based on the significant work on astrocytic control of limbic glutamatergic activity [13] having effects on serotoninergic dorsal raphe function, the role of astrocytes and their heteroreceptor complexes [14,15] is discussed in relation to depression and other mental diseases. Finally, in view of the negative effects of stress on depression development [16] the possible link of stress to effects on 5-HTR heterocomplexes, especially to 5-HT1A auto-receptor complexes in dorsal raphe serotonin neurons, is also briefly discussed.

## 2. D2R-5-HT2AR and D2R-5-HT1AR Heterocomplexes and Mental Disease

### 2.1. D2R-5-HT2AR Heterocomplex

In 2010 two groups [11,12], by means of FRET and BRET methods, respectively, provided the first evidence for the existence of the DA D2R-5-HT2AR heteromer in cellular models. Electrostatic interactions between the third intracellular loop of the D2R protomer and the C-tail of the 5-HT2AR protomer [12] as observed in A2AR-D2R heterocomplexes [17] and triplet amino acid homologies in transmembrane regions [11], appeared to be involved in forming the receptor interface. They also assembled into functionally interacting heteromers. Their existence in the brain was proposed based inter alia on their colocalization in the medial prefrontal cortex [12]. 

It is of interest that hallucinogenic drugs, such as d-Lysergic acid diethylamide (LSD) and those of the indole-alkyl-amine type such as psilocybin, proposed to act as postsynaptic 5-HTR agonists [18,19] turned out to be 5-HTR agonists that mainly target the 5-HT2AR subtype [20]. The effects of the standard 5-HT2AR agonist TCB2 were therefore compared with those of the hallucinogenic 5-HT2AR agonists LSD and 2,5-dimethoxy-4-iodoamphetamine (DOI) on the biochemical binding and signaling of the D2R protomer in the D2R-5-HT2A heteroreceptor complexes. Only the hallucinogenic 5-HT2AR agonists were able to increase the Bmax values of the D2R antagonist binding sites and to increase the affinity of the high-affinity D2R protomer agonist binding sites in the ventral and dorsal striatum and HEK293 cells [21]. The actions of LSD and DOI on D2R affinity and density were blocked by the 5-HT2AR antagonist ketanserin. In the cellular model using a forskolin-induced CRE-luciferase reporter gene assay the hallucinogenic 5-HT2AR agonists significantly enhanced the D2R agonist-induced inhibition of CRE-luciferase activity which likely reflects enhanced inhibitory Gi/o-mediated signaling of the D2R protomer. These enhancing effects by the hallucinogenic drugs were counteracted by ketanserin.

Based on these findings the hypothesis was introduced that hallucinogenic 5-HT2AR agonists at least in part induce their psychotic-like actions by being biased 5-HT2AR agonists at the orthosteric binding site of the 5-HT2AR protomer of the D2R-5-HT2AR heterocomplex, especially located in the ventral striatum. This biased property of the hallucinogenic 5-HT2AR agonists leads to the activation of a facilitatory allosteric mechanism that increases the density and the affinity of the high-affinity component of the D2R protomer, associated with an enhancement of D2R protomer signaling mediated via Gi/o [21,22].

These observations also give a new understanding of the molecular mechanism for the ability of atypical antipsychotic drugs with high affinity for the 5-HT2AR to exert therapeutic effects in psychosis and schizophrenia [23,24]. Most of them are antagonists that have a higher affinity for the 5-HT2AR than for the D2R. Under the assumption that in schizophrenia a pathological enhancement of the facilitatory allosteric receptor–receptor interactions develops in the D2R-5-HT2AR heterocomplexes involving nucleus accumbens [25], these receptor complexes become of high interest as targets in the treatment of schizophrenia. The existence of D2R-5-HT2AR heterocomplexes can help explain the anti-schizophrenic effects of atypical antipsychotic drugs not only based on blockade of 5-HT2AR and D2R in higher doses but also based on blocking the allosteric enhancement of D2R protomer signaling by 5-HT2AR protomer activation. Brain-penetrant hetero-bivalent drugs with 5-HT2AR and D2R antagonist pharmacophores can be therefore of substantial interest by selectively targeting the D2R-5-HT2AR heterocomplexes that can be located in relevant brain circuits such as the ventral striatal-pallidal GABA antireward neurons [26].

It should be considered, however, that each of the classical hallucinogens targeting mainly the 5-HT2AR, such as d-LSD and psilocybin [27] have its own panorama of actions in its modulation of the 5-HT2AR and other types of serotonin heteroreceptor complexes which will have consequences for their mood-modulating effects. It will be an interesting area for the future.

Furthermore, MDMA (3,4-methylenedioxymethamphetamine,“ecstasy”) can exert actions at several subtypes of 5-HT receptors [28], which belongs to the compounds called entactogens. It possesses prosocial effects in animals and may have positive effects in post-traumatic stress disorder [29]. However, DA-serotonin interactions involving increased release of 5-HT and DA are also involved in the actions of MDMA [28]. These early findings support the possibility that one target for the actions of MDMA can be the D2R-5-HT2AR heteroreceptor complex. Thus, D2R-5-HT2AR complexes can represent a center for integration also for the actions of MDMA.

Another feature of interest is that the 5-HT2AR also forms a heteroreceptor complex with oxytocin receptors (OXTR) [30]. The negative effects of 5-HT2AR on depression can involve allosteric antagonistic receptor–receptor interactions in OXTR-5-HT2A heteroreceptor complexes in emotional networks [3]. The inhibition of OXTR protomer signaling by 5-HT2AR can contribute to inhibitory effects on e.g., reward and motivation and disturbances in social behavior [31].

### 2.2. D2R-5-HT1AR Heterocomplex

In 2016 the formation of constitutive D2R-5-HT1AR complexes was demonstrated in cotransfected HEK cells [32]. The method was based on the FRET principle using homogenous time-resolved FRET (HTRF) and fluorescence lifetime imaging microscopy (FLIM). It is of interest that colocalization of the 5-HT1AR and D2R was observed to a high degree in the medial prefrontal cortex (mouse) but to a much lower degree in the striatum and hippocampus based on double immunofluorescence. 

Atypical antipsychotic drugs, especially clozapine, increased the heteromerization of these two receptors in the HEK 293 cell line. Combined treatment with clozapine and the 5-HTR agonist 8-OH-DPAT led to the most marked reduction of cAMP levels that was proposed to involve a 5-HTR agonist-induced allosteric inhibition of the signaling of the D2R [32]. These results again underline the impact D2R-5-HTR heteromerization for the integration of DA and 5-HT signals in the DA and 5-HT nerve terminal networks of the brain, which can be of relevance for understanding schizophrenia and depression and the development of novel treatments.

This work was continued in 2018 [33] with the demonstration of D2R-5-HT1AR heterocomplexes in the prefrontal cortex of the mouse using the in situ proximity ligation assay (in situ PLA). Chronic treatment with the SSRI paroxetine or a low dose of risperidone, an atypical antipsychotic drug with ability to block both 5-HT2AR and D2R, was found to increase D2R-5-HT1AR heteromerization in the prefrontal cortex. This action was found not to be related to an increased expression of these two receptors due to lack of changes in the mRNA levels using in situ hybridization and failure of changes in the density of the D2R and 5-HT1AR using quantitative receptor autoradiography [33]. Thus, the increased D2R-5-HT1AR heteromerization found is independent of changes in expression and may reflect an increased affinity of the two receptors for each other involving recruitment of the D2R and/or 5-HT1AR from monomers, homomers, and other types of D2R and 5-HT1AR heteroreceptor complexes.

It is of substantial interest the demonstration of PLA positive D2R-5-HT1AR heteroreceptor complexes both in the pyramidal glutamate nerve cells and in the GABA neurons, and also in the astroglia, underlining the view that this integrative mechanism is also present in this glial cells [14,15]. It will be of substantial interest to study if these heteroreceptor complexes of high relevance for integration of DA and 5-HT signals are also present in significant densities in other brain regions.

In view of the existence of 5-HT1AR-5-HT2AR heteroreceptor complexes in the hippocampus and anterior cingulate cortex [34] it should be considered that also D2R-5-HT1AR-5-HT2AR heterocomplexes can exist in several regions of the brain in a dynamic balance with D2R-5-HT2AR and D2R-5-HT1AR heteroreceptor complexes and the corresponding homomers and monomers. This research opens a new understanding of the integration of DA and 5-HT signals released from DA and 5-HT nerve terminal networks operating mainly via volume transmission to reach heteroreceptor complexes located in extrasynaptic and synaptic regions of neuronal cells and in astroglia.

## 3. The GalR1-GalR2 Heterodimer and the Putative GalR1-GalR2-5-HT1A Heteroreceptor Complexes. Targets for Galanin N-terminal Fragment Gal (1–15), a Modulator of Emotional Networks in Models of Mental Disease

**Figure 1:**
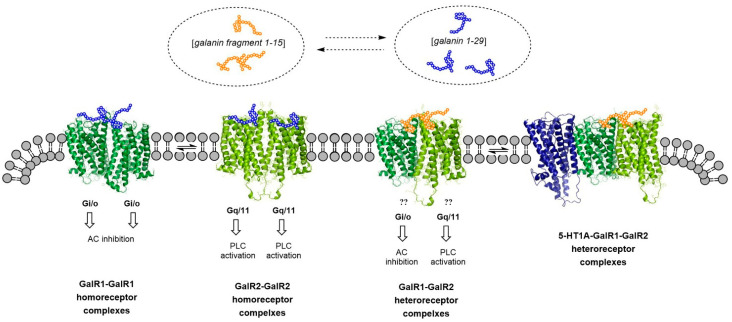
To the left it is shown the location of GalR1 homodimer and the GalR2 homodimer. They are located in the plasma membrane with high affinity mainly for galanin 1–29 (blue circles). GalR1 signals via inhibition of adenylyl cyclase (AC) mediated through Gi/o while GalR2 mainly signals via phospholipase C (PLC) activation mediated through Gq/11. The GalR1-GalR2 heterodimer has instead a high affinity for galanin fragment 1–15 (yellow circles) (to the right). This heterodimer is in equilibrium with the 5-HT1AR-GalR1-GalR2 in the plasma membrane. In this heterotrimeric complex the Gal 1–15 fragment enhances through allosteric mechanisms the 5-HT1AR signaling mainly through activation of GalR2. As a result, antidepressant-like effects were observed upon cotreatment with 5-HT1AR agonist and the galanin fragment given i.c.v. Moreover, an increased expression of the 5-HT1AR protomer was observed.

Gal (1–15) has a high affinity for and preferentially activates the GalR1-GalR2 heterodimer located especially in the raphe-limbic-cortical systems and has a significant role in producing anxiety and depression-related behaviors [35,36]. GalR1activation produces depressive actions while GalR2 stimulation induces antidepressant effects [9]. The reason for the stronger depressive effects of the Gal (1–15) fragment compared with parent galanin peptide [9] may be its ability to preferentially activate with higher affinity and efficacy of the GalR1 protomer vs. the GalR2 protomer of the GalR1-GalR2 complex [37] (Figure 1). This should also lead to increased allosteric inhibition of the GalR2 protomer signaling with its antidepressant activity. 

It should be noted that high-affinity galanin fragment-binding sites were observed also in brain regions with few galanin high-affinity binding sites such as dorsal hippocampus, striatum, and cerebral cortex [36]. In regions with both galanin fragment and galanin-binding sites, the galanin fragment was more potent than galanin in reducing the affinity of the high-affinity 5-HT1AR agonist binding sites [38,39]. In line with these results, only the galanin fragment but not galanin was able to reduce the high-affinity 5-HT1AR agonist binding sites in the hippocampus [40].

In 2016 Millon et al. [8] discovered that Gal (1–15) could enhance the antidepressant-like actions of a 5-HT1AR agonist in the forced swimming test. Furthermore, Flores-Burgess et al. [41] discovered that this Gal fragment improved the antidepressant-like effects of the SSRI fluoxetine in the same test. To explain this switch in the action of Gal (1–15) from causing depression and anxiety to enhancing the antidepressant effects of a 5-HT1AR agonist and the SSRI fluoxetine, the existence of GalR1-GalR2-5-HT1AR complexes was proposed. In this putative complex novel allosteric receptor–receptor interactions can develop. Furthermore both GalR1-5HT1A and GalR2-5-HT1A heteroreceptor complexes exist inter alia in the hippocampus, as demonstrated by in situ PLA analysis [8]. These results are compatible with the formation of GalR1-GalR2-5-HT1AR heterocomplexes in balance with the GalR1-5-HT1AR and GalR2-5-HT1AR heteromers in the hippocampus. 

The mechanism for the ability of the Gal (1–15) fragment to enhance the antidepressant effects of the 5-HT1AR agonist [8] is unknown but it may be that the combined treatment enhances GalR2 signaling via an allosteric mechanism in the putative GalR1-GalR2-5-HT1AR complex. The 5-HT1AR agonist may preferentially enhance the GalR2 protomer signaling which in turn can allosterically inhibit the GalR1 protomer signaling coupled to Gi/o, causing inhibition of the cAMP-PKA signaling pathway and leading to depressive effects. Of high relevance is the coupling of GalR2 to Gq/11 which, upon activation, can increase phospholipase C signaling leading to activation of the calcium signaling pathway. It involves the activation of Erk1/2, p50/p65 and calmodulin which alters gene expression and can cause increases in 5-HT1AR mRNA levels observed as well as the increase of Bmax values of the 5-HT1AR high-affinity agonist binding sites [8]. Such an increase of expression of 5-HT1AR can be a major mechanism involved in the Gal (1–15) induced enhancement of the antidepressant effects of the 5-HT1AR agonist including the raphe-hippocampal 5-HT neurons.

Similar results were also obtained with i.c.v Gal (1–15) in combination with fluoxetine treatment in terms of enhancement of the antidepressant effects of the SSRI using the forced swim test [41]. It is of substantial interest that both, siRNAGalR1 or siRNAGalR2 knockdown in vivo, counteracted the enhancement of these antidepressant-like effects induced by Gal (1–15) in fluoxetine treated rats [41,42]. The involvement of the 5-HT1AR in these events was demonstrated by the blockade of these behavioral effects by a specific 5-HT1AR antagonist (WAY 1000635). These results were compatible with the existence of a GalR1-GalR2-5-HT1AR complex. In line with these results increases in the 5-HT1AR mRNA levels in the dorsal hippocampus, but not in the dorsal raphe, were also observed by the combined treatment.

Another interesting paper was published by Flores-Burgess et al. [43] studying the Gal (1–15)-fluoxetine interactions in the novel object recognition test with participation of 5-HT1AR in the medial prefrontal cortex. This part of the cerebral cortex is a region where emotional processing and cognitive events become integrated and there exist significant densities of GalR1, GalR2, and 5-HT1AR. It is of substantial interest that the fluoxetine-produced memory impairment in the novel object recognition test was reversed by Gal (1–15). The neurochemical mechanism involves the GalR2 subtype, since it was the GalR2 antagonist (M871) which blocked the reversal of fluoxetine effects induced by Gal (1–15) [43]. Other neurochemical findings were that the galanin fragment reduced the affinity and increased the density of the 5-HT1AR agonist binding sites and increased the mRNA levels of the 5-HT1AR 24 h after the treatment.

In order to interpret this fluoxetine-induced memory impairment and its reversal by Gal (1–15), we should consider that there is a balance between the various heteroreceptor complexes in each neuron [2]. In the medial prefrontal cortical neurons, we should consider especially the balance between GalR1-GalR, GalR1-GalR2-5-HT1AR, and 5-HT1AR-5-HT2AR [44] heteroreceptor complexes. 

Furthermore, the heteroreceptor complexes are highly dynamic and it has been proposed that they play a major role in learning and memory [44,45]. In the postsynaptic membranes they can form short-term memories that can be transformed into long-term memories (molecular engram). This can involve a major role for novel adapter proteins formed through the binding of receptor fragments set free from the intracellular part of the receptor protomer to bind and modulate transcription factors. This can allow the expression of novel adapter proteins that can reorganize transient heteroreceptor complex into a long-term memory. Thus, to understand the results obtained by Flores-Burgess et al. [43] we should consider the existence of the GalR1-GalR2-5-HT1AR heterocomplex and its balance with the 5-HT1AR-5-HT2AR heterocomplex in the medial prefrontal cortex. It seems possible that the SSRI fluoxetine increases the extracellular serotonin levels which should activate both 5-HT1AR and 5-HT2AR, with a consequent 5-HT2AR protomer-induced allosteric inhibition of the 5-HT1AR protomer in the 5-HT1AR-5-HT2AR heterocomplex [34]. It should also be considered that the 5-HT2AR monomer and homomer can bind to the 5-HT1AR protomer of the GalR1-GalR2-5-HT1AR heterocomplex and inhibit the signaling also of this 5-HT1AR protomer. Such an inhibition of these two 5-HT1ARs in two different heteroreceptor complexes can help explain the fluoxetine-induced memory impairment in the novel object recognition test in the prefrontal cortex. With the 5-HT1AR signaling inhibited in these two receptor heterocomplexes that can be part of the memory formed together with other high order homo-and heteroreceptor complexes, this memory can no longer function, and novel object recognition memory is lost.

However, the memory could be restored by giving Gal (1–15) together with fluoxetine. The mechanism can involve restoration of 5-HT1AR signaling in the GalR1-GalR2-5-HT1AR heterocomplex. This may be possible because in this heteroreceptor complex Gal (1–15) the allosteric receptor–receptor interactions are different from those in the GalR1-GalR2 complex. Thus, Gal (1–15) may now preferentially activate the GalR2 protomer which via the Gq/11 coupling can activate the signaling of the 5-HT1AR signaling. In addition, the activation of the Gq/11 signaling which increases gene expression can also explain the observed increase in the mRNA levels of the 5-HT1AR. This action will also contribute to increased 5-HT1AR activity [43]. 

It is of interest that Gal (1–15) did not reverse the actions of fluoxetine on the Object Location Memory task [43]. This task likely mainly takes place in the hippocampus and was also impaired by fluoxetine. Thus, it seems as if in this case the GalR1-GalR2-5-HT1AR heterocomplex does not participate in this memory process. Instead, it may involve another 5-HT heteroreceptor complex in which the 5-HTR protomer may need to be reactivated after its down regulation by SSRI fluoxetine activated 5-HTR subtypes potentially with depressive actions.

### Gal (1–15) and Its Modulation of the Meso-Limbic DA Reward Neurons Causes Anhedonia

It is of high interest that Millon et al. [46] found that Gal (1–15) fragment given i.c.v. can cause anhedonia based especially on studies on saccharin self-administration and sucrose preference tests. The neurochemical analysis was focused on the ventral tegmental area, rich in DA cell bodies projecting into the ventral striatum and the cerebral cortex, especially the frontal and limbic cortex, and the nucleus accumbens rich in DA nerve terminals. However, also a substantial number of GABA nerve cells exist in the ventral tegmental area [47].

As previously mentioned, the major target for Gal (1–15) is the GalR1-GalR2 heterocomplex [35,37]. It is therefore of significant interest that the GalR1 and GalR2 were colocalized among the DA and GABA nerve cell bodies of the ventral tegmental area where they form GalR1-GalR2 heterocomplexes. The dominant action of Gal (1–15) at this heterocomplex is usually an activation of the GalR1 protomer with a Gi/o mediated inhibitory signaling and with the ability to allosterically inhibit the GalR2 signaling mediated via Gq/11 which enhances the signaling in the cells.

However, it is important to notice that anhedonia-like behavior, demonstrated e.g., in saccharin self-administration and sucrose preference test after i.c.v. injection of Gal (1–15), was counteracted by the GalR2 antagonist M871 [46]. These results can best be interpreted by assuming that the GalR1-GalR2 heterocomplexes are mainly located on the GABA VTA interneurons innervating the VTA DA neurons. Activation of the GABA interneurons by GalR2 can then lead to inhibition of the VTA DA neurons contributing to anhedonia-like behavior. The blockade of the GalR2 by the antagonist can then cause a return of the activity in the VTA-limbic-cortical DA neurons which counteracts the anhedonia-like behavior [46]. As to a leading role of GalR2 vs. the GalR1 protomer in this regulation of the VTA DA neurons, it was found that Gal (1–15) reduced the mRNA expression of the DA transporter and vesicular monoamine transporter 2 that also reflect an increased GABA inhibition of the VTA DA neurons. Instead, there was a significant increase in the mRNA expression of D3R in the VTA by Gal (1–15) which can be explained by the fact that the D3R can operate mainly as an auto-receptor in VTA [46]. The results are compatible with the view that the VTA GalR2 protomer upon activation by Gal (1–15) induced an activation of the GABA interneurons causing inhibition of the VTA DA neurons leading to anhedonia-like behavior and a neurochemical down regulation of the meso-limbic DA reward neurons [46]. These results are supported by the observations of a reduction of the mRNA levels of DAT and vesicular monoamine transporter 2 after Gal (1–15) treatment. This should lead to increased activation of the D2 and D3 auto-receptors producing a reduced neuronal activity in the VTA-Limbic-Cortical DA neurons [46].

In line with these results, reduced tyrosine hydroxylase immunoreactivity was found in the nucleus accumbens as well as reduced mRNA levels of C-Fos [46]. Thus, signs of a down-regulation of the VTA-Limbic-Cortical DA system also appeared to develop in the nucleus accumbens after treatment with Gal (1–15). As a compensatory upregulation, significant increases of the mRNA levels of DA D1R, D2R, and D3R were observed in the nucleus accumbens in response to the reduced activity induced in these DA neurons by Gal (1–15) [46].

This work by Millon et al. [46] indicates that GalR1-GalR2 heterocomplex activated by Gal (1–15) may directly activate the GABA interneurons in the VTA leading to inhibition of the VTA-Limbic-Cortical DA pathway which produces anhedonia-like behavior, a major sign of depression. It seems possible that the previous work reporting strong depression, based on testing in the forced swim test, and anxiety with i.c.v. Gal (1–15) [35] also can involve at least in part activation of GABA interneurons also in regions such as the hippocampus and dorsal raphe. This will be an important continuation of the work on understanding the mechanism of action of Gal (1–15) besides the participation of the GalR1-GalR2 heteromer in higher order heteroreceptor complexes with the 5-HT1AR and enhancing its signaling [2,10,36].

The remarkable down-regulation of the activity of VTA-Limbic-Cortical DA reward neurons observed with Gal (1–15) with a postulated dominance of GalR2 activation give thoughts to the idea that GABA interneurons in the VTA upon activation has a major role in putting a brake on the VTA-limbic-Cortical DA pathway to reduce reward and introduce anhedonia. It should be considered that this regulation should have an impact not only in major depression but also for the negative symptoms of schizophrenia. It will also be of high interest to study if Gal (1–15) via its GalR1-GalR2 heteromer has a similar role in regulating the GABA interneurons which innervate the nigro-striatal DA neurons. 

## 4. Serotonin 5-HT1AR-Receptor Tyrosine Kinase (RTK) Heteroreceptor Complexes

**Figure 2:**
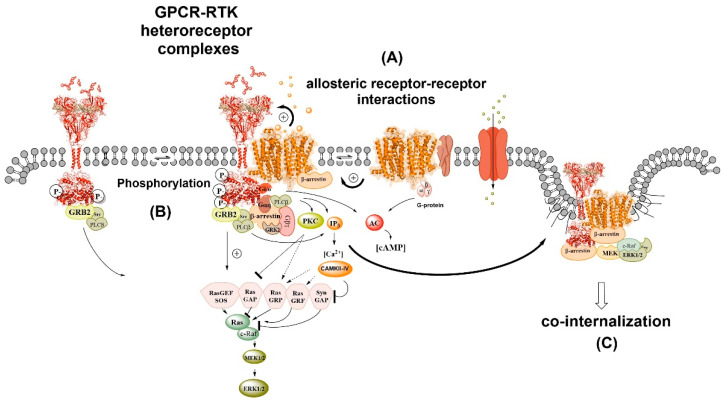
GPCR-RTK heteroreceptor complexes exemplified by the A2A-TrkB heteroreceptor complex [51]. To the left the TrkB homodimer is found in the plasma membrane signaling via growth factor receptor-bound protein 2 (GRB2), proto-oncogene tyrosine protein kinase (src) and PLC beta. At the center, in the plasma membrane, it is shown the A2AR homodimer which is found interacting with an adapter protein (e.g., RAMP) and its link to the Gs alpha, leading to the enhancement of cAMP levels via activation of the AC. At the center, it is also shown the A2A-TrkB heteroreceptor complex and their allosteric receptor–receptor interactions (**A**) which take place between the A2AR protomer and TrkB receptor protomer enhancing the TrkB receptor signaling. The TrkB receptor signaling can also be enhanced through phosphorylation (**B**) involving transactivation via e.g., activation of src. A number of signaling proteins are indicated in cytoplasm below the heteroreceptor complex, leading via various Ras proteins to activation of MEK1/2 and ERK1/2. To the far right, the co-activation of the two receptor protomer in the heteroreceptor complex leads through recruitment of beta-arrestin to co-internalization, reducing the signaling of the heteroreceptor complex (**C**).

In 2007, it was proposed that direct GPCR-RTK interactions through the formation of GPCR-RTK heteroreceptor complexes can lead to transactivation of the RTK protomer independent of metalloprotease activation to transform a pro-ligand into a RTK ligand to elicit RTK signaling [48,49]. Inspired by the significant work of Kitayama et al. [50] we proposed that certain postjunctional 5-HT1R subtypes may activate the fibroblast growth factor receptor FGFR2-FGFR1 system by forming a heteroreceptor complex leading to development of allosteric receptor–receptor interactions (Figure 2). In this way it seemed possible that SSRI, such as fluoxetine, may improve also neural trophic activity and counteract depression-induced atrophy of the serotonin raphe-limbic neuron systems through transactivation of FGFR1 via allosteric mechanisms and indirect interactions over intracellular pathways. It was also proposed that transactivation of RTK may also develop via activation of the intracellular pathways of GPCRs involving increases of intracellular calcium ions, protein kinase Src, and messengers such as reactive oxygen species which cause tyrosine phosphorylation of the RTK [49].

The first evidence for this view was obtained by Flajolet et al. [52], who demonstrated that FGF can act as a co-transmitter through adenosine A2AR to regulate synaptic plasticity. Using the yeast two hybrid analysis physical interactions were demonstrated through the formation of A2AR-FGFR heteroreceptor complexes. Coactivation of these two receptor protomers caused in PC12 cells neurite extension and MAPK-ERK activation and induction of cortical-striatal plasticity, opening up new possibilities for drug development [52].

In 2012, evidence was obtained for the existence of FGFR1-5-HT1AR heteroreceptor complexes in the raphe-hippocampal 5-HT system [3,7,53]. These heteroreceptor complexes were discovered in hippocampal cultures and in the dorsal hippocampus using co-immunoprecipitation and in situ PLA [15,53]. A neuronal location was observed and upon coactivation of the two receptor protomers, synergistic increases in extracellular signal-regulated kinases 1 and 2 were found. The highest density of FGFR1-5-HT1AR heterocomplexes were present in the pyramidal cell layer of the CA1 to CA4 areas mainly modulating their glutamate projections and their plasticity. The enhancement of hippocampal plasticity also involved synergistic increases in PC12 cell neurite densities and extensions as well as protrusions in hippocampal cultures. These plasticity changes were blocked by interference with the receptor interface of the heteromer. It was of high interest that the acute and combined treatment with FGF-2 and 5-HT1AR agonist produced marked antidepressant-like effects as demonstrated by using the forced swim test. 

In 2015 evidence was obtained also for the existence of FGFR1-5-HT1AR autoreceptor complexes in the dorsal and median raphe of the midbrain projecting inter alia into the hippocampus [54]. Enhancement of the FGFR1 protomer signaling was observed with improved trophic effects also in the FGFR1-5-HT1A autoreceptor complexes upon combined agonist treatment [55]. The neurophysiological work of Ambrogini et al. (2021) led to the remarkable demonstration that agonist activation of the FGFR1 protomer led to a substantial reduction of the 5-HT1A autoreceptor function through an allosteric inhibition of the 5-HT1A-induced opening of the G protein-coupled inwardly rectifying potassium (GIRK) channels. In this way the hyperpolarization of the 5-HT nerve cells becomes reduced, improving neuronal activity in the ascending 5-HT neuron systems leading to antidepressant activity.

## 5. Other Types of GPCR-RTK Heterocomplexes and Treatment of Depression

### 5.1. GPCR-TrkB Heterocomplexes

This year the exciting discovery was made that a number of antidepressant drugs such as tricyclic antidepressants (TCAs) and SSRIs, as well as the fast-acting antidepressant drug ketamine, can bind to the TrkB receptor [56]. The binding site for fluoxetine was shown to be in the transmembrane domain of the TrkB heterodimer. The binding of the antidepressants to this site enhanced the behavioral actions of BDNF including their positive effects on neuronal plasticity. Another highlight was that mutation of the transmembrane domain counteracted the behavioral actions of the antidepressant drugs. The link to the effects of the antidepressant drugs to enhanced neuronal plasticity was suggested to explain the slow development of the antidepressant-like effects of antidepressant drugs. Previous work strongly indicated that the signaling of BDNF was essential for the effects of almost all antidepressant drugs [57,58]. However, the study of Casarotto et al. [56] now demonstrated that antidepressant drugs can directly bind to the TrkB homo-dimer in the transmembrane domain with an affinity that is relevant for obtaining therapeutic effects. The analysis involved atomistic molecular dynamics simulations. It was also found that the transmembrane domain of the TrkB heterodimer can sense alternations in the levels of cholesterol in the plasma membrane, which can be of relevance for its ability to mediate plasticity [56]. Cholesterol is derived from astroglia and modulates BDNF signaling and BDNF enhances neuronal synthesis of cholesterol [59].

The above results from Casarotto et al. [56] are of high interest and demonstrate that antidepressant drugs through targeting a novel binding site in the transmembrane part of the TrkB homodimer can allosterically promote the signaling of BDNF over the TrkB receptor. This integrative process can be further developed through the existence of GPCR-TrkB heteroreceptor complexes in diverse brain regions as discussed above. The GPCRs can then, especially upon agonist activation allosterically modulate the BDNF-TrkB signaling in the GPCR-TrkB heteroreceptor complex. So far, only the A2AR-TrkB heterocomplex in the striatum has been demonstrated [51]. It will be of high interest to test if a discrete number of 5-HTR subtypes can form heteroreceptor complexes with the TrkB receptor. Upon activation of the 5-HT receptor protomer the allosteric receptor–receptor interactions may then enhance the signaling of the TrkB receptor homodimer and promote neuronal plasticity and antidepressant-like behavioral activity in animal models of depression. 

It is of high interest that very recently Madhusmita et al. [60] published a paper on the effects of depletion of TrkB receptors located in adult 5-HT neurons. They found clear-cut modulations of the neurochemistry and function of the 5-HT neurons. They found marked increases in the brain 5-HT levels and likely in 5-HTsynthesis and release and deficiency in learning and memory together with improved energy metabolism. These results open the possibility that the TrkB homodimer receptor can form a heteroreceptor complex with the 5-HT1A autoreceptor in the 5-HT neurons, in which the TrkB protomer can enhance the 5-HT1A protomer affinity for 5-HT and 5-HT1A autoreceptor signaling based on facilitatory allosteric receptor–receptor interactions. As a result of enhanced inhibitory 5-HT1A autoreceptor activity, the firing rate of the 5-HT neurons will decline through the enhanced opening of the GIRK channels increasing hyperpolarization.

Based on the loss of activation of the 5-HT1A autoreceptor by the TrkB receptor in this heteroreceptor complex, it seems possible to understand the reason for the putative overactivation of 5-HT synthesis and release upon deletion of TrkB receptors in the adult 5-HT neurons. It should also be noticed that 5-HT1A autoreceptor-FGFR1 heterocomplexes exist in the 5-HT neurons [6,54]. It will be of high interest to evaluate if these heterocomplexes have a differential role in modulating the activity and plasticity of the dorsal raphe 5-HT neurons via their 5-HT1A autoreceptors and to understand their balance in the adult 5-HT neurons.

It is of substantial interest that Di Palma et al. [51] demonstrated the A2AR-TrkB heteroreceptor complexes in the dorsal hippocampus also have their highest density in the pyramidal cell layer including the CA1 to CA3 regions and the polymorphic layer of the dentate gyrus. Thus, different types of GPCR-RTK hetero complexes appear to exist to a high degree in the pyramidal cell layer of the hippocampus giving it substantial neuronal plasticity necessary for the learning and memory activities ongoing in the glutamate pyramidal neurons of these regions. This high plasticity likely becomes impaired as aging develops leading to cognitive disturbances [51]. Brain-derived neurotrophic factor (BDNF) is the ligand for the TrkB receptor and deficits in its production may be one factor to be considered when discussing the malfunction of learning and memory in aging and overall errors in the allosteric receptor–receptor interactions in the GPCR-RTK heterocomplexes are likely to play a major role.

Angiotensin receptor type 2 can form heteroreceptor complexes with TrkB receptors in the medial prefrontal cortex leading to transactivation of TrkB. Moreover, an antagonist of the angiotensin receptor produces antidepressant effects [49]. These results open the possibility that this heterocomplex can be a novel target for antidepressants [61]. It should also be noticed that activation of 5-HT7 receptor promotes TrkB receptor expression and phosphorylation in primary cultures from the cerebral cortex [62]. However, these effects may not be linked to antidepressant actions since this 5-HT receptor subtype possesses depressive activity. However, this may not be true when existing in a complex with TrkB. It is of substantial interest that Rantamaki et al. [63] found that antidepressant drugs such as imipramine can transactivate the TrkB receptor in the rodent brain independently of BDNF and blockade of monoamine transporters. The mechanism remains to be determined but it may potentially involve the possible existence of certain monoamine receptor-TrkB heteroreceptor complexes. In this case, the extracellular monoamines can induce conformational changes in monoamine receptor protomers that via allosteric receptor–receptor interactions can contribute to the transactivation of the TrkB protomer. As a result, increases in neuronal survival and plasticity may develop.

### 5.2. Muscarinic Acetylcholine Receptor (mAChR)-FGFR1 Hetero Complexes

Muscarine M3R-FGFR1 heterocomplexes were found in the hippocampus using in situ PLA [64]. Subsequently the existence of M1R-FGFR1 heterocomplexes was explored in the dorsal hippocampus [65]. M1R-FGFR1 heterocomplexes were found with receptor–receptor interactions leading to a rapid transactivation of the FGFR involving allosteric modulation. The transactivation was connected to neuronal plasticity since neurite outgrowth was counteracted by the pFGFR inhibitor and the Src inhibitor. With PLA the hetero receptor complexes were observed inter alia in the pyramidal cell layer of the hippocampus. The M1R protomer can therefore take part in the FGFR transactivation. The allosteric receptor–receptor interactions in these complexes demand the phosphorylation of the FGFR by receptor and nonreceptor tyrosine kinases to improve FGFR signaling [65]. It should also be considered that nuclear FGFR1 may operate as a general transcriptional regulator [66].

Treatments involving pFGFR inhibitor SU5402 or Src inhibitor PP1 on FGFR transactivation induced by a mAChR agonist indicated a mechanism in which potential allosteric receptor–receptor interactions between FGFR1 and mAChR depends on the Src protein linked to these heteroreceptor complexes. It suggested that the allosteric mechanisms in the heteroreceptor complexes demand a correct recruitment and activation of the Src tyrosine kinase protein for the AChR agonist to produce the transactivation of the hippocampal FGFR1. Thus, a tyrosine kinase of the Src protein family is crucial for the operation of the allosteric receptor–receptor interaction [65]. It enables the muscarinic receptor activation of the trophic FGFR1 system with modulation of differentiation, growth, and protection of neurons.

It should also be considered that the mAChR subtypes in the hippocampus are located in a pre and post-synaptic position and can participate in higher order heteroreceptor complexes, also containing FGFR1 protomers and ionotropic receptors. Through their modulation the mAChR1,3 subtypes can have a significant role in learning and memory [44,65]. The mAChR1,3-FGFR1 heterocomplex may also be a new target for treatment of Alzheimer’s disease by reducing neurodegeneration upon its activation, including the malfunction of the synaptic heteroreceptor complexes.

Moving into mental disease, it was found by Veena et al. [67] that the mAChR agonist oxotremorine could improve depression-like behavior in chronically stressed rats. Furthermore, it seems more than possible that depression-induced atrophy in hippocampal neurons can be improved by treatment with muscarinic agonists, especially in combination with brain penetrant FGFR subtype specific agonists [28,65]. Based on the work of Borroto-Escuela et al. [53,68] (see above) a combined treatment with 5-HT1AR agonists and muscarinic1,3 receptor agonists would be of high interest in view of the existence of both 5-HT1AR-FGFR1 and mAChR1,3-FGFR1 heterocomplexes in the hippocampal neurons, especially of the pyramidal cell layer. These heteroreceptor complexes may be in balance with 5-HT1AR-FGFR1-mAChR1,3 hetero complexes, should they be expressed in the same neurons.

### 5.3. GPCR-Epidermal Growth Factor Receptor (EGFR) Heterocomplexes

EGFR belongs to the ErbB family of tyrosine kinase receptors and become functional through homodimerization and heterodimerization in a ligand-dependent way [69]. It possesses neuromodulation and neurotrophic activities [49]. It is of substantial interest that adenosine A1 receptors can produce transactivation of the EGFR leading to neuroprotective actions in cortical neurons [70]. These two receptors are co-located in the plasma membrane of nerve cells supporting the view that A1R-EGFR heterocomplexes may be formed. However, they remain to be demonstrated.

Another GPCR, the D2R can upon its activation increase the degree of co-immunoprecipitation supporting the view that D2R activation can increase the formation of this putative heteroreceptor complex in striatal neurons [71]. The D2R protomer appears capable of producing a transactivation of EGFR protomer through stimulation of a non-receptor tyrosine kinase (Src) leading to the activation of extracellular signal-regulated kinase (ERK) signaling. The D2R can have a relevant role in neuroprotective effects and in the development of the DA neuron systems.

It should also be noticed that GPCR-EGFR complexes may also be formed in astroglia but this remains to be demonstrated. Upon activation of 5-HT2BR in astroglia via fluoxetine-induced increases in extracellular levels of 5-HT, transactivation of the EGFR can develop followed by ERK phosphorylation and EGF shedding [72]. Via volume transmission neuronal EGFR can also become activated by EGF and may also contribute to the antidepressant effects of fluoxetine. It is also of substantial interest that activation of the 5-HT2 receptor causes Src-dependent FGFR2 transactivation followed by ERK and CREB phosphorylation and expression of GDNF mRNA in glial cells [73]. It should be considered that multiple mechanisms can be involved in the GPCR transactivation of EGFR-like tyrosine phosphorylation and allosteric receptor–receptor interactions.

### 5.4. Do GPCR-Platelet-Derived Growth Factor Receptor (PDGFR) Heteroreceptor Complexes Exist?

PDGFs and their ligands have a major role in the development of the nervous system and its maintenance [74]. So far, no GPCR-PDGFR heteroreceptor complexes have been reported. Of special interest is the demonstration that 5-HT and 5-HT1AR agonists can increase the phosphorylation of the PDGF beta in cortical neurons and neuroblastoma cells. This transactivation turned out to be Src and PLC dependent [75,76]. Furthermore, the SSRI fluoxetine could transactivate the PDGFR beta via activation of 5-HT2B receptors which blocked the 5-HT-induced transactivation likely through desensitization [77]. Both D2R and D4R were capable of transactivating PDGFR beta via tyrosine phosphorylation. Furthermore, it is also of significant interest that the D2R and D4R-induced transactivation of PDGFR beta can lead to inhibition of NMDAR signaling in the pyramidal cell layer in the hippocampus and in the prefrontal cortex involving a calcium ion and calmodulin-dependent NMDAR inactivation [78]. In 2006 it was demonstrated that the D2R can inhibit NMDAR signaling via formation of a D2R-NMDAR complex [79]. Thus, the regulation by D2R of NMDAR may involve in certain brain regions higher-order heteroreceptor complexes in which D2R-PDGFR beta-NMDAR can participate. Such heteroreceptor complexes may indicate that not only GPCRs but also RTKs can modulate synaptic transmission and thus have an impact on learning and memory through modulation of the composition of the homo-and heteroreceptor complexes, their allosteric receptor–receptor interactions, and their phosphorylation and dephosphorylation via striatal-enriched protein tyrosine phosphatase (STEP). In this way short-term memories and long-term memories and thus molecular engrams can be formed in the postsynaptic membrane of synapses [44,45].

## 6. Can the Biological Principle of Forming Heteroreceptor Complexes in the Brain Help Understand the Mechanism of Action of Hallucinogens?

### 6.1. MDMA (3,4-Methylenedioxymethamphetamine)

MDMA belongs to the family of entactogens and is known for its prosocial actions and was shown to be able to improve the symptoms of post-traumatic stress disorder [28,80,81,82]. MDMA was found to produce multiple neurochemical effects such as inhibitory actions on the DA, noradrenaline (NA) and 5-HT transporters and increases the blood levels of oxytocin, cortisol, and vasopressin. It can directly bind to certain 5-HT receptor subtypes, mainly to 5-HT1AR and 5-HT2B-C receptors. These receptors, such as the vasopressin 1A receptor activated by oxytocin, may be involved in the prosocial effects of MDMA [81]. The real targets that induce the prosocial effects of MDMA and its therapeutic effects in post-traumatic stress disorder is unknown. In view of the existence of e.g., 5-HT1AR-5-HT2AR, 5-HT2AR-OXTR, and 5-HT2CR-OXTR heteroreceptor complexes [2,31,83], it seems possible that such and other 5-HT heteroreceptor complexes can be high-affinity targets for MDMA actions. These heteroreceptor complexes can also become modulated by activated glucocorticoid receptors in stress, known to be enriched in the monoamine neurons [84].

### 6.2. Psilocybin

This psychoactive alkaloid belongs to the classic 5-HT hallucinogens [84] also called psychedelic compounds [85]. Its major target appears to be the 5-HT1A, 5-HT2A, and 5-HT2C receptors [85]. There exist promising indications that psilocybin can produce therapeutic effects in depression [86]. It is proposed that the major target of psilocybin can be certain 5-HTR heterocomplexes in which due to conformational changes certain 5-HTR heterocomplexes have developed a high affinity for psilocybin as well as a high efficacy leading to enhanced signaling of this 5-HTR subtype. Such an effect can also be region selective due to the differences in their composition. There is the possibility that such heteroreceptor complexes can mediate fast antidepressant effects, but there is no support for this possibility. Such events may also develop with the 5-HT hallucinogen dimethyltryptamine, which can activate several subtypes of 5-HT receptor besides the 5-HT2AR, known to play a major role in mediating the hallucinogenic actions [82].

### 6.3. Ketamine

Ketamine is a rapidly acting antidepressant drug used in treatment of resistant depression. It is hallucinogenic but belongs to the dissociative anesthetics [82]. Its mechanism of action has not been fully clarified but it has been suggested that the antidepressant actions of ketamine may involve a noncompetitive antagonist action at certain NMDARs and activation of the mammalian target of rapamycin 1 (mTORC1) pathway [87]. However, the early work of Saarelainen et al. [88] should be also noted. They demonstrated that the positive effects of antidepressant drugs such as ketamine were counteracted by blocking TrkB signaling. Castren and Antila [58] therefore proposed that the antidepressant effects were produced by TrkB-induced increases in neuronal plasticity.

The recent work of Casarotto et al. [56] is highly interesting since they found that not only classical antidepressant drugs such as fluoxetine but also ketamine and its metabolite can bind to the TrkB receptors in concentrations that do not block the NMDA receptor. These exciting results suggest that both TCA and SSRI antidepressant drugs as well as the rapidly acting antidepressant drug ketamine can produce significant therapeutic effects through binding and acting at partially overlapping sites in the transmembrane domain of the TrkB receptor in critical neuronal networks [56]. They also proposed that the more rapid antidepressant actions of ketamine can be explained by its more rapid penetration into the brain reaching more quickly the concentration needed for the activation of TrkB.

## 7. Understanding the Role of Astrocytes in Modulating Forebrain Glutamate Synapses with Impact on Dorsal Raphe-Forebrain Serotonin Neurons Playing a Key Role in Major Depressive Disorder

### 7.1. Key Features of Astrocytes

It is today well-known that the astrocytes possess a large number of receptors, especially those belonging to the GPCR family [89]. As examples can be mentioned monoamine [90], adenosine, and mGluR5 receptors among the GPCRs. Astrocytes can release transmitters especially glutamate, D-serine being a co-agonist at the NMDA receptor and ATP which is hydrolyzed to adenosine in the extracellular space [91]. Via short distance volume transmission [92,93] the astroglial transmitters such as glutamate released by astroglial Ca2+ signals can directly reach the neuronal glutamate receptors leading to increases in neuronal Ca2+ signals as well [94,95,96].

It is true that the astrocytes are not electrically excitable but are excitable in terms of Ca2+ oscillations found through the use of confocal and 2-photon microscopy as well as fluorescent indicators of Ca2+ (see [97]). These Ca2+ oscillations can be propagated as calcium waves in astrocytes through astrocytic release of ATP [98]. Thus, astrocytes can encode information using Ca2+ signaling.

It appears clear that astrocytic mGluR5 can play a significant role in increasing the degree of astrocytic Ca2+ signaling. This glutamate receptor is Gq coupled which activates phospholipase C leading to increased formation of diacylglycerol and inositol triphosphate (IP3), which can release Ca2+ from its stores in the astrocytes and increase the Ca2+ levels in the cytoplasm. It should also be considered that astroglia via release not only of glutamate but also of D-serine, can regulate neuronal NMDA receptors since D-serine acts as a co-agonist at the glycine binding site of the NMDA receptor. Thus, it is clear that astrocytes in this way can modulate synaptic glutamate plasticity [99]. 

When discussing the role of astrocytes, it should be noted that an important source of glutamate originates from the astrocytic glutamate-glutamine cycle (see [100]). It involves the uptake of glutamate into astrocytes via their astroglial transporters followed by its binding to an astrocytic enzyme, glutamine synthetase leading to the formation of glutamine. Upon release of glutamine from the astrocytes it is taken up into neurons by transporters. Here glutaminase converts glutamine into glutamate. In the GABA neurons glutamate then becomes decarboxylated into GABA and stored in vesicles. It documents the major role of astrocytes in synaptic GABA transmission.

Finally, when discussing the function of the astrocytes the marked impact of interactions between astrocytes and neurons on brain energy metabolism should be remembered [101]. There is a need not only for neurovascular coupling but also for neurometabolic coupling involving demands of cooperation between activated astrocytes and neurons to meet high energy demands to develop appropriate neuronal excitability and function. Of special interest is lactate in the brain that is to a substantial degree formed and released from astrocytes. Lactate reaches neurons via volume transmission and acts not only by providing energy but also by providing signals to the neurons [102]. Magistretti and Allaman [102] regard lactate as a modulator of neuronal function to improve excitability and plasticity to allow the development of “an optimal homeostatic tone.” It represents an exciting achievement.

### 7.2. Astroglial Heteroreceptor Complexes 

**Figure 3:**
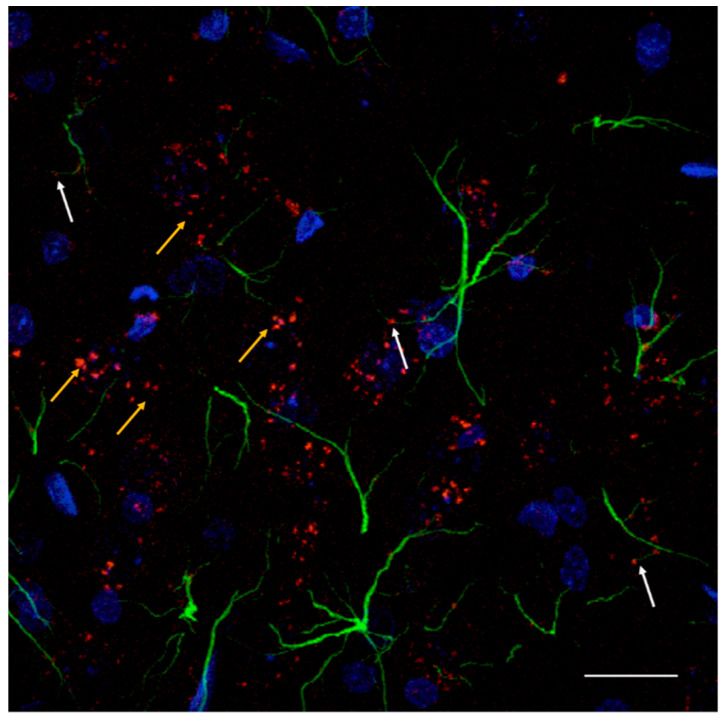
In the nucleus accumbens shell A2AR-D2R heteroreceptor complexes were demonstrated both in astrocytes and in nerve cells. The astrocytes were visualized in green color with GFAP antibodies (glial fibrillary acidic protein immunoreactivity). The nerve cells were not visualized. PLA positive A2AR-D2R hetero complexes were found as clusters in high densities. White arrows point to the red PLA clusters in green labeled astroglia. Most of the red clusters are not associated with the astroglia (yellow arrows) and are most likely present in the accumbal neurons rich in GalR1 and GalR2 and 5-HT1ARimunoreactivities.

In 2017 the first indications were obtained that allosteric receptor–receptor interactions can exist in astrocytes [14]. Thus, A2AR-D2R interactions were shown to modulate gliotransmitter release from striatal astrocytes. The confocal analysis in isolated striatal astrocytes demonstrated their co-location. Furthermore, the activation of D2R inhibited the 4-aminopyridine (blocker of potassium channels that delays repolarization) induced release of glutamate from the astrocytes and this action was blocked by A2AR activation [14]. The A2AR agonist alone lacked effects on the astroglial glutamate release. These results were compatible with the existence of antagonistic allosteric A2AR-D2R interactions also in astroglial A2AR-D2R hetero complexes. This view was strongly supported by the demonstration that the synthetic D2R peptide VLRRRRKRN present in the intracellular loop 3, which is part of the electrostatic interface with the C- tail of the A2AR, can effectively block the inhibitory action of the A2AR agonist on D2R-induced inhibition of astroglial glutamate release by 4-aminopyridine [14]. In a subsequent paper biochemical and biophysical evidence was obtained for the existence of A2AR-D2R hetero complexes on striatal astrocytes based on the use of coimmunoprecipitation and proximity ligation assay [103] (Figure 3). Thus, this important integrative mechanism present in heteroreceptor complexes exist also in astroglia as beautifully demonstrated in these two publications.

It should also be underlined that FGFR1-5-HT1AR heteroreceptor complexes exist in hippocampal astrocytes which may have a role in the 5-HT and FGF2 modulation of hippocampal gamma oscillations [15]. The pharmacological analysis indicated the existence of antagonistic allosteric receptor–receptor interactions in the astroglial FGFR1-5-HT1AR heterocomplexes that can contribute to diminishing the 5-HT1AR-induced reduction of gamma oscillations [15]. These results further underline the role of heteroreceptor complexes as a significant integrative mechanism also in astrocytes.

### 7.3. On the Role of Astrocytes in the Control of Glutamatergic Activity in the Infralimbic Cortex Modulating the Activity of the Ascending Raphe-Limbic-Cortical Systems

Gasull-Camos et al. [104] made the exciting discovery that the blockade of the astroglial transporter GLT-1 in the infralimbic cortex of rats can produce rapid antidepressant-like actions. Previous work had indicated that ketamine can elicit fast antidepressant actions via AMPA receptor (AMPAR) activation in the medial prefrontal cortex [105]. A similar AMPAR activation in the infralimbic cortex likely takes place also after the blockade of GLT-1 through microinfusion of the GLT-1 inhibitor dihydrokainic acid leading to enhanced astroglial glutamate release and activation of AMPAR. Thus, the effects were mimicked by an AMPA receptor agonist s-AMPA and blocked by an AMPAR antagonist.

This group also emphasized the infralimbic cortex connections to the midbrain raphe serotonin neurons projecting into the forebrain and having a highly significant role in major depressive disorder [5,106]. It is of high interest that microinfusion of veraridine, a depolarizing compound, into the infralimbic cortex produced rapid antidepressant actions. The mechanism likely involved the activation of the serotonin raphe-cortical neurons, via the descending glutamate pathways from the infralimbic cortex, supported by the demonstration that the antidepressant-like effects produced by the astroglial transporter were no longer induced, following serotonin synthesis inhibition. Instead, the SSRI citalopram given into the infralimbic cortex had antidepressant effects similar to those of the astroglial transport blocker [104]. 

The serotonin mechanisms involved in the antidepressant-like effects of astrocytic transporter blockade with dihydrokainic acid in the infralimbic cortex, were further studied in 2018 [107]. It is of substantial interest that local 5-HT1ARs mediated the antidepressant effects of dihydrokainic acid and citalopram in the infralimbic cortex since they were blocked by the microinjection of the 5-HT1ARs antagonist WAY100635 in this region. Nevertheless, it was found that the astroglial transporter inhibition in the infralimbic cortex increased serotonin and glutamate release in the dorsal raphe of the midbrain. In support, increased expression of c-Fos was demonstrated in the serotonin nerve cells of the dorsal raphe but not in its GABA interneurons [107] and increased serotonin release was also found in the ventral hippocampus. 

It should be noted that inhibition of the astrocytic transporter in the infralimbic cortex versus the AMPAR agonist action gives a more prolonged duration of the antidepressant effects. It may be related to a longer duration and enhanced intensity of the glutamate drive from the pyramidal neurons of the infralimbic cortex after astrocytic transporter inhibition. It can be due to a prolonged release of glutamate from the astrocytes enhancing the activation of multiple extrasynaptic and synaptic glutamate receptors on the infralimbic cortical glutamate neurons to the dorsal raphe. The increased 5-HT release induced in the dorsal raphe by the glutamate drive can also activate the 5-HT1A autoreceptor and contribute to its down regulation in the serotonin neurons of the dorsal raphe increasing the duration and enhancement of activity in the midbrain-limbic-cortical serotonin pathway [107].

These studies especially underline the impact of direct glutamate connections activating the dorsal raphe serotonin neuron from the infralimbic cortex to produce antidepressant-like effects involving projections of the dorsal raphe neurons also into the ventral hippocampus showing increased serotonin release. However, also locally activated postjunctional 5-HT1ARs in the infralimbic cortex play a role in mediating antidepressant actions of locally given SSRI and inhibitors of astrocytic glutamate transport.

### 7.4. Selective Knockdown of Astrocytic Glutamate Transporters in the Infralimbic Cortex. Depressive-Like Phenotypes in Mice

In contrast to the acute pharmacological blockade of astroglial glutamate transporters above in the infralimbic cortex, a selective knockdown of these transporters in the same region led to a depressive phenotype in mice [108]. These experiments, leading to a stronger activation of the descending glutamate pathway to the dorsal raphe and with a longer duration were performed, since it was proposed that disturbances in the astrocytes of the ventral anterior cingulate cortex (infralimbic cortex in rodents) together with increased energy metabolism participate in producing major depressive disorder (MDD). Knockdown was produced by microinjecting small interfering RNA, targeting the GLT-1 or BLAST astrocytic glutamate transporters, into the infralimbic cortex [108]. Reductions in the number of glutamine synthase and GFAP positive astrocytes were observed linked to the development of a depressive phenotype. This state could be reversed by ketamine and SSRI.

The substantially increased glutamate drive to the dorsal raphe from the infralimbic cortical region after knockdown of the astroglia glutamate transporters was demonstrated in neurophysiological experiments on pyramidal neurons in layer V [109]. An increased firing rate of action potentials was observed together with membrane potentials that were more depolarized. Spontaneous excitatory postsynaptic currents with elevated amplitude and frequency were also found. However, this highly enhanced glutamate drive to the dorsal raphe region led to a marked reduction of serotonin release in the dorsal raphe that likely led to a strong inhibition of the activity in the meso-limbic-cortical serotonin pathways globally innervating these regions [110]. The deficit in serotonin transmission can help explain the reductions of the BDNF that can explain the reduced structural and functional plasticity found in MDD [108]. Of interest is also their observations of an increased formation of early growth response protein 1 (Egr-1) in the activated infralimbic cortical glutamate neurons to the dorsal raphe after the knockdown of the astroglial glutamate transporter. This may directly improve GABAA receptor expression, composition, and maintenance [111] in the dorsal raphe serotonin projection neurons upon Egr-1 release from the activated infralimbic cortical glutamate neurons onto these serotonin neurons and cause their enhanced inhibition. It can be a way to keep the balance of glutamate excitation and GABA inhibition to reach a proper synaptic strength. However, in the current case a pathological inhibition of the dorsal raphe serotonin neurons was obtained.

In addition, highly significant studies [14,109,110] clearly indicates that the degree of glutamate drive activation to the dorsal raphe induced by astrocyte glutamate transporter inhibition or knockdown have a major impact on whether the dorsal raphe serotonin neurons become activated or inhibited. In their exciting work they have pointed out that an important factor is also whether the activated glutamate projection neurons from the infralimbic cortex mainly target the local GABA neurons inhibiting the dorsal raphe serotonin neurons causing a depressive phenotype or mainly directly activate the dorsal raphe serotonin neurons with their rapid activation producing fast antidepressant actions. This work has put the astrocytes and their interactions with serotonin neurons into a top position in the field of depression and its treatment together with serotonin and other types of heteroreceptor complexes.

## 8. Conclusions and Future Work

The formation of heteroreceptor complexes in the brain appears to represent a fundamental integrative mechanism in the brain that can be disturbed in mental disease leading to pathological disturbances in neuronal-astrocyte networks especially in the limbic system and its connections with the lower brain stem. The focus of this review was on the 5-HT heteroreceptor complexes and their role in major depression. There exist many types of 5-HT heteroreceptor complexes in the limbic system and highly relevant 5-HT1A autoreceptor complexes exist in the dorsal raphe neurons sending their axons into the forebrain and diencephalon. It is concluded that 5-HT receptor subtypes physically interact not only with other GPCRs such as DA receptor subtypes and Gal R subtypes but also with several types of RTKs such as TrkB and FGFR1, allowing direct integrative interactions also in the modulation of plasticity and neuroprotection that can involve also the astroglia. It is also of high interest that Casarotto et al. [56] have demonstrated that antidepressant drugs can directly bind to the TrkB receptors. Therefore, for the future it will be of interest to find out if the TrkB heteroreceptor complexes can be of special interest for understanding major depression development involving the integration of 5-HTR and TrkB receptor signaling through allosteric and phosphorylation/di-phosphorylation processes. In the future work it will also be of high interest to further develop the work of Gasull-Camos et al. [104] on the role of astrocytes in the control of glutamate activity in the infralimbic cortex modulating the activity of the raphe-forebrain 5-HT neurons. This is also true for the work of Fullana et al. [108] showing depression-like phenotype in mice after a selective knockdown of the astrocytic glutamate transporter in the infralimbic cortex.
